# THP1 proteomics in response to *mycobacterium tuberculosis* infection

**DOI:** 10.1016/j.dib.2021.106803

**Published:** 2021-01-30

**Authors:** Ajay Kumar, Mukul K. Midha, Kanury VS Rao

**Affiliations:** a Translational Health Science and Technology Institute, NCR Biotech Science Cluster, Faridabad, Haryana, India; b Institute for Systems Biology, Seattle, WA 98109, USA

**Keywords:** THP1, Mycobacterium tuberculosis, Temporal, Proteomics, Mass spectrometry

## Abstract

Temporal data on how the mycobacterium infection establishes itself inside the host cell is not available. We differentiated human THP1 cell line with PMA and infected them with different laboratory (H37Ra and H37Rv) and clinical strains (BND433 and JAL2287) of mycobacterium tuberculosis (Mtb). Uninfected differentiated THP1 cells were used as infection control. Host proteome was investigated at four different time points to understand the dynamics of host response to mycobacterial infection with time. The investigated time points included 6 hrs, 18 hrs, 30 hrs and 42 hrs of infection with all the Mtb strains. SWATH-MS method was used to quantitate the host proteome in response to Mtb infection and the data thus obtained are available via PRIDE repository with the dataset identifier PXD022352 (https://www.ebi.ac.uk/pride/archive/projects/PXD022352).

## Specifications Table

**Table:** 

Subject	Infectious Diseases
Specific subject area	Perturbations in host macrophage proteome in response to infection with *Mycobacterium tuberculosis*
Type of data	Table
How data were acquired	Reverse-phase high-pressure liquid chromatography electrospray ionization tandem mass spectrometry (RP-HPLC-ESI-MS/MS) using a NanoLC-Ultra 1D plus (Eksigent; Dublin, CA) and nanoFlex cHiPLC system (Eksigent) which is directly connected to an ABSCIEX 5600 Triple-TOF (AB SCIEX; Concord, Canada) mass spectrometer.
Data format	Raw
Parameters for data collection	Proteomics alteration in PMA differentiated THP1 cell line was captured subsequent to infection with H37Ra, H37Rv, BND433 and JAL2287 strains of mycobacterium tuberculosis. Un-stimulated host proteome was collected in uninfected THP1 cells. Using SWATH-MS method proteomics data on host response after 6, 18, 30 and 42 hour of mycobacterial infection was collected.
Description of data collection	Host proteome from uninfected as well as MTb infected THP1 cells were investigated in triplicates at four time points. Peptides identified at 1.0% Global FDR from Fit (Figure 1) were quantified using a spectral library generated in-house. 50 femtomoles β-galactosidase was injected with each MS run as an internal standard and was used for auto-calibration of spectra after acquisition of every injection using dynamic LC-MS and MS/MS acquisitions.
Data source location	Translational Health Science and Technology Institute, NCR Biotech Science Cluster, Faridabad, Haryana, India
Data accessibility	Repository name: ProteomeXchange Data identification number: PXD022352 Direct URL to data: https://www.ebi.ac.uk/pride/archive/projects/PXD022352/

## Value of the Data

• Temporal proteomics data provides insight into how the mycobacterium establishes within the host to use its machinery for personal survival.
• This data will be useful to understand dynamics of host response to mycobacterial infection.
• This data can be used to identify host proteins specific to stage of mycobacterial infection to further understand host pathogen interactions.
• The difference in quantitative dynamics of host proteins showing common and/or unique response to infection with laboratory and clinical mycobacterial strains can be delineated using this dataset.
• THP1 proteome in infected as well as uninfected conditions can be compared.

## 1 Data Description

**Fig. 1:**
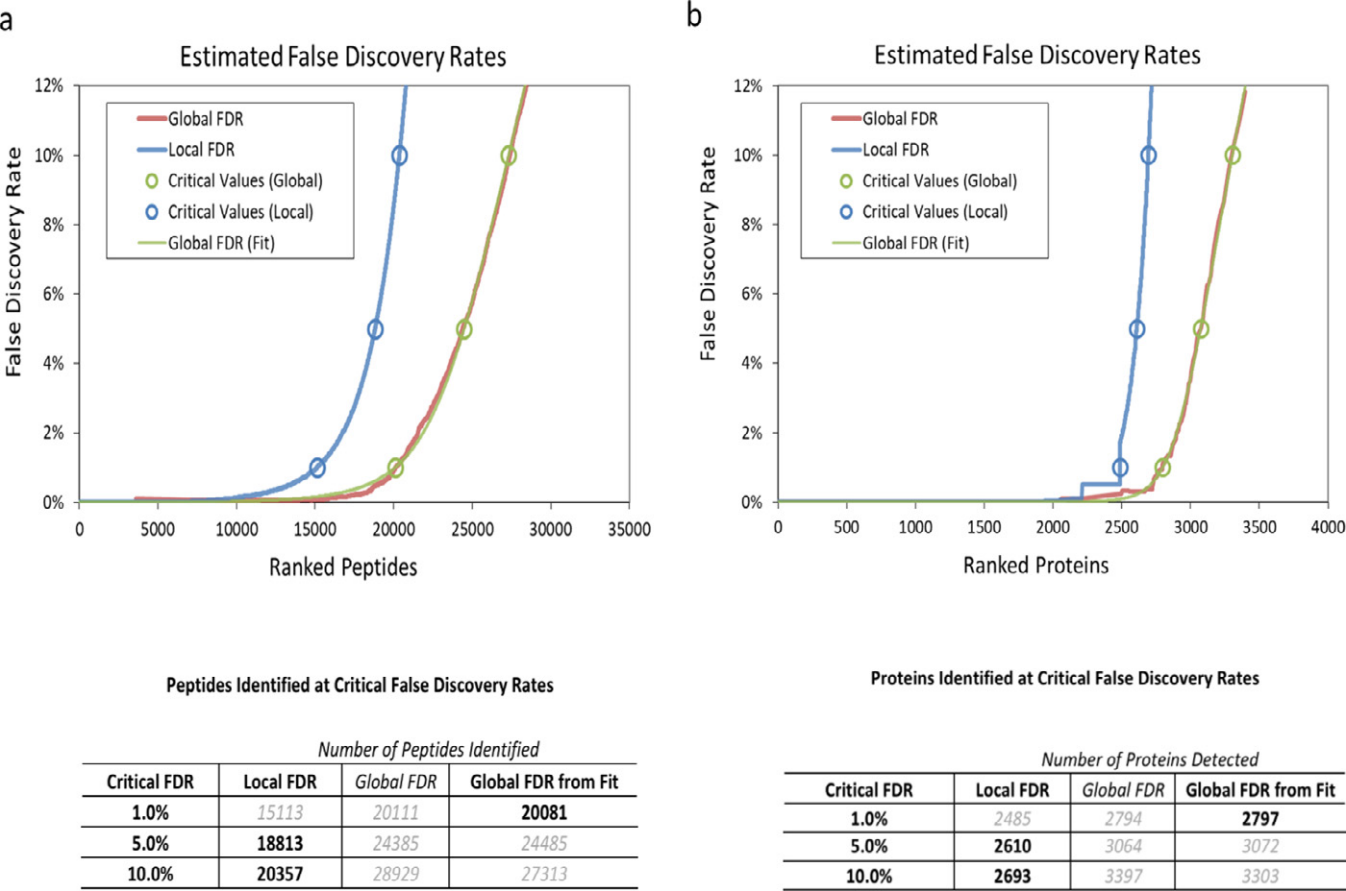
The measured False Discovery Rates analysis for (a) peptides, (b) proteins in human THP1 sample-specific assay library generation using SCIEX ProteinPilot.

In this dataset, we have captured changes in proteome of human macrophage-like cells (THP1 cell line) after infection with mycobacterial strains. PMA differentiated THP1 cells were infected with mycobacterial strains of varying virulence (two laboratory strains – H37Ra and H37Rv and two clinical strains – BND433 and JAL2287) and host proteome was monitored over multiple time points up to 42 h of infection. Cells were differentiated in parallel sets to ensure similar conditions for all Mtb strains and time points and bacteria were added at 10 multiplicity of infection. After infection, host cells were collected and lysed at 6, 18, 30 and 42 h and the proteome was investigated using SWATH-MS method against uninfected THP1 cells. A total of 20,081 peptides were identified at 1.0% Global FDR from Fit (Fig. 1). The detected proteins (*n* = 2797) provide important insights into temporal dynamics of the host response to different strains of Mtb.

Two supplementary tables have been provided in support of our data manuscript. Supplementary Table 1 is a THP1 spectral assay library generated from DDA-MS measurements in Protein Pilot/Paragon search engine. Supplementary Table 2 is a quality assessment report by, DIALib-QC (DIA library quality control) in-house developed software tool at ISB, which provides systematic evaluation of the THP1 assay library's coverage, characteristics, completeness and correctness across 62 important parameters.

## 2 Experimental Design, Materials and Methods

### 2.1 Cell culture

Middlebrooke 7H9 broth (Difco) supplemented with 10% ADC (Becton Dickinson), 0.4% glycerol and 0.05% Tween 80 was the medium of choice for Mtb cultures. Bacteria were collected by centrifugation in mid-log phase and later re-suspended in RPMI medium. Aspirating ten times through 23-guage and then a 26-gage needle to prevent bacterial clumping dispersed bacterial cultures. An additional 5 times dispersion step followed this through a 30-gage needle. Bacterial count was determined using upper half suspension of the dispersed cultures by spectrophotometric analysis (at 600 nm wavelength an optical density value of 0.6 would correspond to approximately 100 million bacteria).

Culture conditions for THP1 cell line were as described by ATCC. Briefly, RPMI 1640 (Gibco) supplemented with 10% FCS (Hyclone) and 1x penicillin (stock concentration = 100 U/ml) and streptomycin (stock concentration = 100 μg/ml) were used to culture the cell line. Cells were maintained in complete media at 37 °C in humidified conditions with 5% CO2. Trypan blue was used to confirm cell viability.

### 2.2 Infection with mycobacteria

THP1 cells were differentiated into macrophage-like cells in presence of 5 ng/ml PMA for 48 h. A total of 30 million THP1 cells per T-175 flask were differentiated in multiple flasks to accommodate all time points and strains. Mtb count was determined and 10 bacteria per THP1 cell were added to initiate infection. Un- infected cells were maintained in parallel culture as control. After infection, THP1 cells were trypsinized and collected at 6, 18, 30 and 42 h by centrifugation at 4 °C. Cells were lysed on ice in presence of benzonase (Sigma) and 2x Protease inhibitor cocktail (Pierce) in Urea lysis buffer (8 M Urea, 200 mM Tris pH8, 4% CHAPS, 1 M NaCl) by sonication (5 - 6 pulses for 3 s each). Total cellular protein concentration was determined by Bradford method. Protein samples were digested with trypsin and prepared for LC-MS/MS as reported before [1].

### 2.3 Strong cation exchange

Peptides were separated in first dimension by cation exchange (SCX) chromatography. The pooled and dried peptides were reconstituted in 1 mL Buffer A (5 mM ammonium formate, 30% (v/v) ACN and 0.1% FA pH= 2.9). These were then added onto a SCX cartridge (5 µm, 300 Å bead from Sciex, USA) with a cartridge holder (Sciex, USA) using a hand syringe set. Sample fractions were collected through a step gradient of increasing ammonium formate concentration (50 mM, 80 mM, 100 mM, 150 mM, and 250 mM ammonium formate, 30% v/v ACN and 0.1% formic acid; pH= 2.9).

### 2.4 Nano-LC-mass spectrometry analysis—

(A) **Data-dependent acquisition (DDA) mass spectrometry**

For spectral library generation, peptide mixtures from each fraction were analyzed on a SCIEX quadrupole time-of-flight (TOF) TripleTOF 5600 mass spectrometer (SCIEX, Concord, CAN) coupled to a NanoSpray II ion Source (SCIEX) and interfaced with nanoLC-Ultra ID plus (Eksigent, Toronto, Canada). Reverse Phase - HPLC were performed via an elute configuration and the peptide samples were separated using one MonoCap C18 High Resolution 200 cm (LCGC Sciences, Japan) long column set up. The 1x iRT reference peptides (Biognosys AG, Schlieren, Switzerland) and 200 femtomol β-galactosidase (as internal standard) were added to all fractions prior to MS injection for retention time calibration. To ensure optimal sample delivery reproducibility, the auto-sampler was operated in full injection mode overfilling a 1 μl loop with 3 μl analyte. The LC-system was operated with 2% ACN, 0.1% formic acid (FA) in water (buffer A) and 0.1% FA in 98% CAN (buffer B) at a flow rate of 400nL/min. The separation was achieved using a linear gradient from 5–50% of buffer B over the period of 275 min. The column was regenerated by a 40 min wash with 90% buffer B and re-equilibrated for another 45 min with 5% buffer B. The instrument was auto calibrated after every sample injection using dynamic LC–MS and MS/MS acquisitions of 50 femtomol β-galactosidase. The MS was operated in DDA mode in DDA top 20 mode with the following parameters: MS1 spectra were collected at 350–1250 *m/z* for 200 ms, 20 most intense precursor per cycle with charge state of 2–5 that exceeds 100 counts/s were selected for fragmentation using activated rolling collision energy, dynamic exclusion was set to 12 s, and the corresponding fragmentation MS2 spectra were collected at 200–1800 m/z for 70 ms.

(B) **Data-independent acquisition (DIA) SWATH- mass spectrometry**

SWATH-MS acquisitions were operated using NanoLC-Ultra 1D plus (Eksigent, Toronto, Canada) and nanoFlex cHiPLC system (Eksigent) were used in direct connection to an ABSCIEX 5600 Triple-TOF (AB SCIEX; Concord, Canada) mass spectrometer [2]. The peptide samples were separated at 35  °C using two Nano cHiPLC columns (Eksigent) in tandem to achieve higher resolution and better separation in chromatography. The analytical column (75 μm × 15 cm) was manufacturer (Eksigent)-packed with 3 μm ChromXP C-18 (120 Å). The buffers used for separation and the injection volume per sample is same as described above in DDA-MS section. For a time point, each sample was reconstituted in 7 μl of buffer A supplemented with 1x iRT and 100 femtomol β-galactosidase. Each fraction was measured in replicates from the analytical column at a flow rate of 400 nL/min by using linear gradient from 5–50% of buffer B over the period of 180 min. The column regeneration and re-equilibration were done by washing with 90% buffer B for 20 min and with 5% buffer B for 40 min respectively. A 10 μm SilicaTip electrospray PicoTip emitter (New Objective Cat. No. FS360–20–10-N-5-C7-CT) was used to inject peptides into the mass spectrometer. The ion source was operated with following parameters: ISVF = 2000; GS1 = 25; CUR = 25. To achieve better specificity in complex samples in SWATH MS-based experimental samples, the instrument was tuned to specifically allow smaller precursor isolation windows in Analyst v1.7 acquisition software (SCIEX). A 57-variable-window setup was generated using the SWATH® Variable Window Calculator 1.1 (SCIEX) with a 1 m/z window overlap on the lower side of the window.  The MS1 survey scan was acquired from 350–1250 m/z for 200 ms and MS2 spectra were acquired in high-sensitivity mode from 200–1800 m/z for 60 ms. The total cycle time was ∼3.6 s. Ions were fragmented for each MS/MS experiment in the collision cell using rolling collision energy.

### 2.5 Generation of sample specific spectral library

The sample specific spectral library was constructed using Protein Pilot software v. 5.0 (AB SCIEX, Revision- 4769) with in-built Paragon algorithm. The raw DDA data files were searched using a through identification effort against a non-redundant, UniProt Swiss-Prot canonical homo sapiens protein sequence database (January 2015 release), appended with the Biognosys iRT peptides and β-galactosidase FASTA sequences. The alkylation reagent was iodoacetamide, with carbamidomethyl (C) as a fixed modification, and oxidation (M), as variable modification, and digestion enzyme was set to Trypsin. Peptides confidence score of > 0.05 was set as a cut-off for further analysis. To generate a high-quality spectral assay library, a false discovery rate (FDR) analysis was performed. The output of this search is a .group file that was used directly for generation of in-house sample specific spectral library using PeakView v.2.1 (Sciex). The .group file includes information required for targeted data extraction: protein name and UniProt accession, cleaved peptide sequence, modified peptide sequence, relative intensity, precursor charge, unused Protscore, confidence, and decoy result. The FDR threshold parameters used to identify and quantify the proteins are described in detail in our previous work [3]. For the FDR analysis, a threshold of 1% accepted Global False discovery rate (G-FDR-fit) criteria was selected for identification and quantification of peptides and proteins. It corresponds to identification of 20,081 peptides and 2776 distinct proteins (green line) in the sample-specific assay library (Fig. 1 and Supplementary Table 1). At each time point, samples from two biological replicates for each Mtb strain (H37Ra, H37Rv, JAL & BND) were analyzed and the time course data identified of whole cell human proteins.

### 2.6 Spectral assay library quality control using DIALib-QC

The in-house THP1 spectral assay library was evaluated using DIALib-QC [4], a freely available software tool that highlights a library's complexity, characteristics, modifications, completeness and correctness available at http://www.swathatlas.org/DIALibQC.php. The DIALib-QC assessment report of the assay libraries is provided in Supplementary Table 2.

### 2.7 Spectral alignment and targeted data extraction

For the spectral alignment and extraction of the targeted data, datasets from SWATH MS/MS acquisitions would be processed using the full scan MS/MS filtering module for DIA within PeakView v.2.1 (Sciex) and Marker view v.1.2.1 (Sciex) software using the In-house sample specific spectral library. The iRT peptides were used for spectral retention time alignment between in-house library and swath data datasets. All SWATH raw files were loaded and exported in .txt format using an extraction window of 10 min and with the following parameters: upto 3 peptides per protein, 6 transitions per peptide, peptide confidence of >95%, 10% G-FDR, exclude shared peptides, and XIC width would be set at 50 ppm. This export would result in the generation of three distinct files containing the quantitative output for (1) the area under the intensity curve for individual ions, (2) the summed intensity of individual ions for a given peptide, and (3) the summed intensity of peptides for a given protein. This protein data would be used for all data analysis in the Marker view software. The reversed sequences were removed from the data set prior to further analysis.

## Ethics Statement

Not Applicable.

## CRediT Author Statement

**Ajay Kumar:** Conceptualization, Methodology, Cell culture, Infection experiments, Sample generation, Analysis, Writing, and Editing; **Mukul K. Midha:** Methodology, Sample preparation, Software, Data Acquisition, Analysis, Writing, Editing; **Kanury VS Rao:** Conceptualization, Supervision.

## Declaration of Competing Interest

The authors declare that they have no known competing financial interests or personal relationships, which have, or could be perceived to have, influenced the work reported in this article.

## Appendix Supplementary materials

Image, application 1

Image, application 2

## Footnotes

Supplementary material associated with this article can be found in the online version at doi:10.1016/j.dib.2021.106803.
